# Factors affecting the early establishment of neonatal intestinal flora and its intervention measures

**DOI:** 10.3389/fcimb.2023.1295111

**Published:** 2023-12-01

**Authors:** Guangyu Ma, Yuguo Shi, Lulu Meng, Haolong Fan, Xiaomei Tang, Huijuan Luo, Dongju Wang, Juan Zhou, Xiaomin Xiao

**Affiliations:** ^1^ Department of Obstetrics and Gynecology, The First Affiliated Hospital of Jinan University, Guangzhou, China; ^2^ National Key Laboratory of Science and Technology on Advanced Composites in Special Environments and Center for Composite Materials and Structures, Harbin Institute of Technology, Harbin, China

**Keywords:** newborn, intestinal flora, caesarean section, premature delivery, breast milk

## Abstract

In recent years, it has become evident that early-life intestinal flora plays a pivotal role in determining human health. Consequently, it is imperative to explore the establishment of neonatal intestinal flora and its influencing factors. Early neonatal intestinal flora is influenced by a multitude of factors, including maternal and infant-related factors, as well as external environment. This review summarizes the colonization mechanism of intestinal flora in the early life of newborns and discussed their influence on the establishment of neonatal intestinal flora, taking into account factors such as delivery mode, gestational age and feeding mode. Additionally, this review delves into the natural or artificial reconstruction of intestinal flora colonization defects in infants born via cesarean section and premature infants, with the goal of establishing a theoretical foundation for preventing and treating issues related to neonatal intestinal flora colonization and associated diseases.

## 1 Introduction

The establishment of the human intestinal microbiota during early life plays a critical role in determining human, exerting profound effects on metabolic processes, growth, immune function, and even behavioral outcomes (Clemente et al., 2012). In recent years, there has been a substantial body of research focused on exploring the composition and functionality of the infant gut microbiota, revealing a distinct correlation between reduced microbial diversity and aberrant structural composition of the gut microbiota and the development of various diseases that may manifest in infancy or later in life, including conditions such as asthma, inflammatory bowel diseases, and metabolic disorders (Milani et al., 2017). Consequently, it is imperative to comprehensively investigate the governing principles and contributing factors that shape the establishment of the neonatal gut microbiota during early life. With the rapid advancements in metagenomic sequencing technology, it is possible to explore the intricate internal patterns and interrelationships within the highly complex gut microbiota. In this review, we have undertaken a comprehensive review of the research progress pertaining to the colonization mechanisms of the early-life intestinal microbiota in newborns. We have delved into the extensive discussion of its profound influence on the establishment of the neonatal intestinal microbiota, considering critical factors such as delivery mode, gestational age, and feeding practices. Furthermore, we have conducted a rigorous analysis of both natural and artificial strategies for rectifying colonization deficiencies within the intestinal microbiota, specifically in the context of infants born via cesarean section and preterm birth. Our primary objective is to furnish a well-founded theoretical framework that can inform and guide proactive measures for the prevention and treatment of intestinal microbiota colonization anomalies and the resultant diseases affecting clinical infants.

## 2 Establishment of early intestinal flora

### 2.1 Discussions about the origin of intestinal flora colonization——utero or during childbirth

The traditional view suggests that the fetal environment *in utero* is sterile, and the colonization of the intestinal flora commences at birth. During childbirth, the neonate first encounters maternal intestinal, vaginal, and skin flora, as well as the surrounding environment. This initial contact facilitates the colonization and proliferation of the intestinal flora, which gradually evolves and establishes a mature microflora structure postnatally (Adlerberth, 2008). It is evident that maternal symbiotic bacteria play a crucial role in the early-life establishment of the neonatal intestinal flora. However, in line with pertinent research (Jiménez et al., 2005; DiGiulio et al., 2008; Aagaard et al., 2014; Collado et al., 2016; Chu et al., 2017b), bacteria were found in the placenta, umbilical cord and amniotic fluid in full-term pregnancy, which suggests that the fetus may not be entirely sterile *in utero* and that the initial colonization of the fetal intestinal flora may indeed commence within the maternal uterus. Jimenez E (Jiménez et al., 2005) conducted tests on umbilical cord blood flora from neonates delivered by cesarean section, revealing the presence of bacteria belonging to *Enterococcus*, *Streptococcus*, *Staphylococcus*, and *Propionibacterium*. Additionally, in animal experiments, the intestines of pregnant mice were inoculated with genetically labeled *enterococcus faecium*, and the labeled *enterococcus faecium* was detected in the feces of young mice delivered by cesarean section, suggesting the potential transmission of maternal microorganisms to the fetus *in utero* (Jiménez et al., 2008). The first meconium of a newborn can reflect the fetal situation *in utero*, and bacteria can be detected in the first meconium of a newborn (Kennedy et al., 2021; Turunen et al., 2021). These investigations provide compelling evidence supporting the concept of fetal intestinal flora colonization initiating *in utero*.

However, several studies have raised questions regarding whether fetal gut flora colonization truly initiates *in utero*. For instance, Kennedy KM (Kennedy et al., 2021) reported that they did not detect bacteria in the meconium collected from healthy newborns born via cesarean section. This suggests that, although microbial presence was observed in the fetal meconium for the first time, the majority of fetuses were delivered vaginally. The meconium collection typically occurs several hours to a few days after birth, and the microflora found in meconium may colonize during delivery or after birth. Therefore, it is plausible that the microflora found in meconium might colonize during delivery or in the postnatal period. Some studies have suggested an absence of flora in the amniotic fluid and placenta. RCR technology was conducted on the amniotic fluid of 344 women from 15 to 22 weeks of pregnancy, and the results showed that mycoplasma, bacteria and fungi in the amniotic fluid samples were negative (Rowlands et al., 2017). The amniotic fluid samples of 10 women who had cesarean section were tested, and the test results showed that the bacterial load in the amniotic fluid was no different from that in the blank control group (sterile Nacl), but the bacterial load in the amniotic fluid samples of 14 women with rupture of fetal membrane was 10 times higher than that of those who had cesarean section (Rehbinder et al., 2018). Amniotic fluid remains sterile due to the protective membrane. For infants with premature rupture of the membrane, intestinal flora colonization may initiate *in utero*, and fetal intestinal flora colonization may begin after the rupture of the membrane. Additionally, 16S rRNA sequencing technology was used to examine placenta-like flora in 29 women who did not give birth to full-term infants via cesarean section, and the results showed that there was no significant difference in the composition and structure of the bacterial spectrum between the placenta samples and the control group (Theis et al., 2019). Some scholars have raised concerns that bacterial flora determination technology primarily relies on bacterial 16S rRNA gene amplification, and the reagents and DNA extraction kits used may be susceptible to contamination from environmental bacteria. Such contamination could potentially affect the accuracy of bacterial flora determination and may not fully reflect the actual bacterial flora in the sample (Mühl et al., 2010; Salter et al., 2014). There is still a lot of controversy about whether fetal intestinal flora colonizes *in utero* or after delivery.

### 2.2 Origin of intestinal microflora in infants

**Figure 1:**
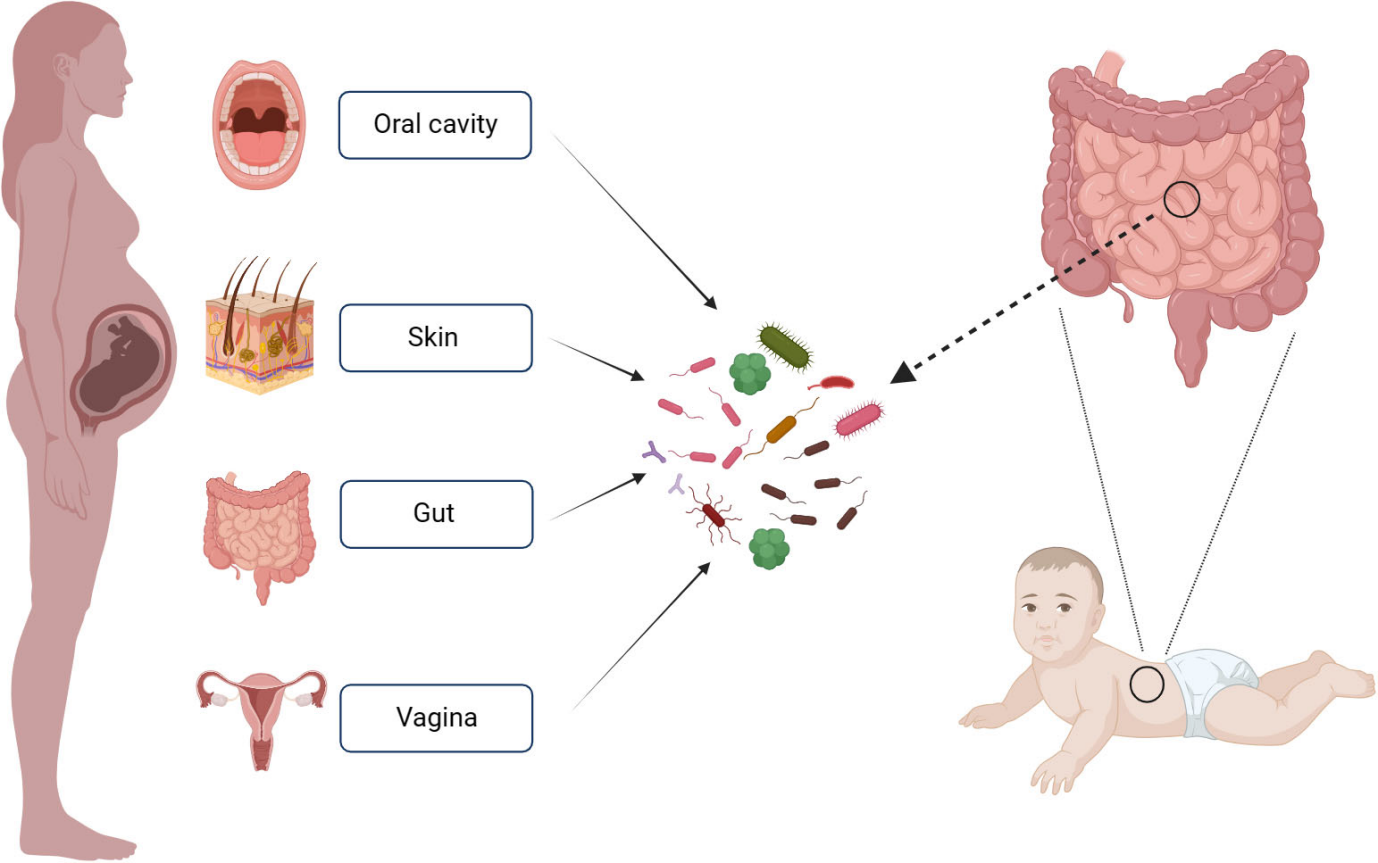
Maternal Sources of Neonatal Gut Microbiota. This figure was created with BioRender.com.

To investigate the origin of intestinal microflora in infants and the contribution of various maternal bacterial flora from different body sites to the establishment of infant intestinal microflora, Ferretti P and colleagues (Ferretti et al., 2018) collected the bacterial samples from the mother’s gut, vagina, skin and oral cavity. The results revealed that bacterial flora from these maternal sites contributed to the establishment of infant’s intestinal microflora. Maternal origins of neonatal gut microbiota are depicted in **Figure 1**. Notably, the maternal fecal microbiome accounted for the largest proportion of the contribution at 22.1%, followed by vagina (16.3%), mouth (7.2%) and skin (5%). Over time, the contribution of the maternal fecal microbiome increased, while the contributions from the vagina, mouth, and skin decreased. The colonization of the infant’s intestinal tract by the mother’s vaginal, oral, and skin flora was relatively transient. Interestingly, species from the maternal vaginal flora constituted approximately 3.5% of the total fecal microbial population in infants at 3 days after birth, but they were no longer detectable in the infant’s feces by the first week. This phenomenon may be attributed to the differences between the vaginal and intestinal environments, as the maternal vaginal strains faced challenges in establishing a lasting presence in the infant’s gut. The infant’s intestinal environment. Given the transient nature of maternal vaginal colonization in the infant’s gut, the feasibility and potential benefits of “vaginal seeding” warrant further discussion.

The microbial environment characterized by aerobic or facultative anaerobic bacteria, such as *Enterobacter*, *Enterococcus* and *Staphylococcu*s, is established in the early stages of a newborn’s intestine. These bacteria consume oxygen during their growth, thereby altering the intestinal microenvironment to favor the proliferation of anaerobic bacteria, including *Bifidobacterium*, *Clostridium*, and *Bacteroides* (Adlerberth, 2008). Within the first week of birth, the infant’s intestinal flora undergoes changes influenced by the mother’s vaginal and skin flora. The maternal intestinal flora becomes increasingly dominant in colonizing the newborn’s intestinal tract. The alpha diversity of the initial flora decreases initially but gradually increases from the first to the third month of life. During this period, the infant’s microflora is primarily composed of *Bifidobacterium* and *Bacteroides*. It’s noteworthy that a stable gut microbiota resembling that of adults is typically established between the ages of 2 to 4 years (Stewart et al., 2018; O’Neill et al., 2020). Thus, neonates undergo a developmental process to establish their intestinal flora. In the initial stage, the intestinal flora is relatively simple and fragile, characterized by low species diversity, rendering it susceptible to external factors. Over time, however, the intestinal flora becomes relatively stable. This underscores the complexity of gut microbiota changes, with our current understanding representing only a fraction of the larger picture.

## 3 Influencing factors of neonatal intestinal flora establishment

**Figure 2:**
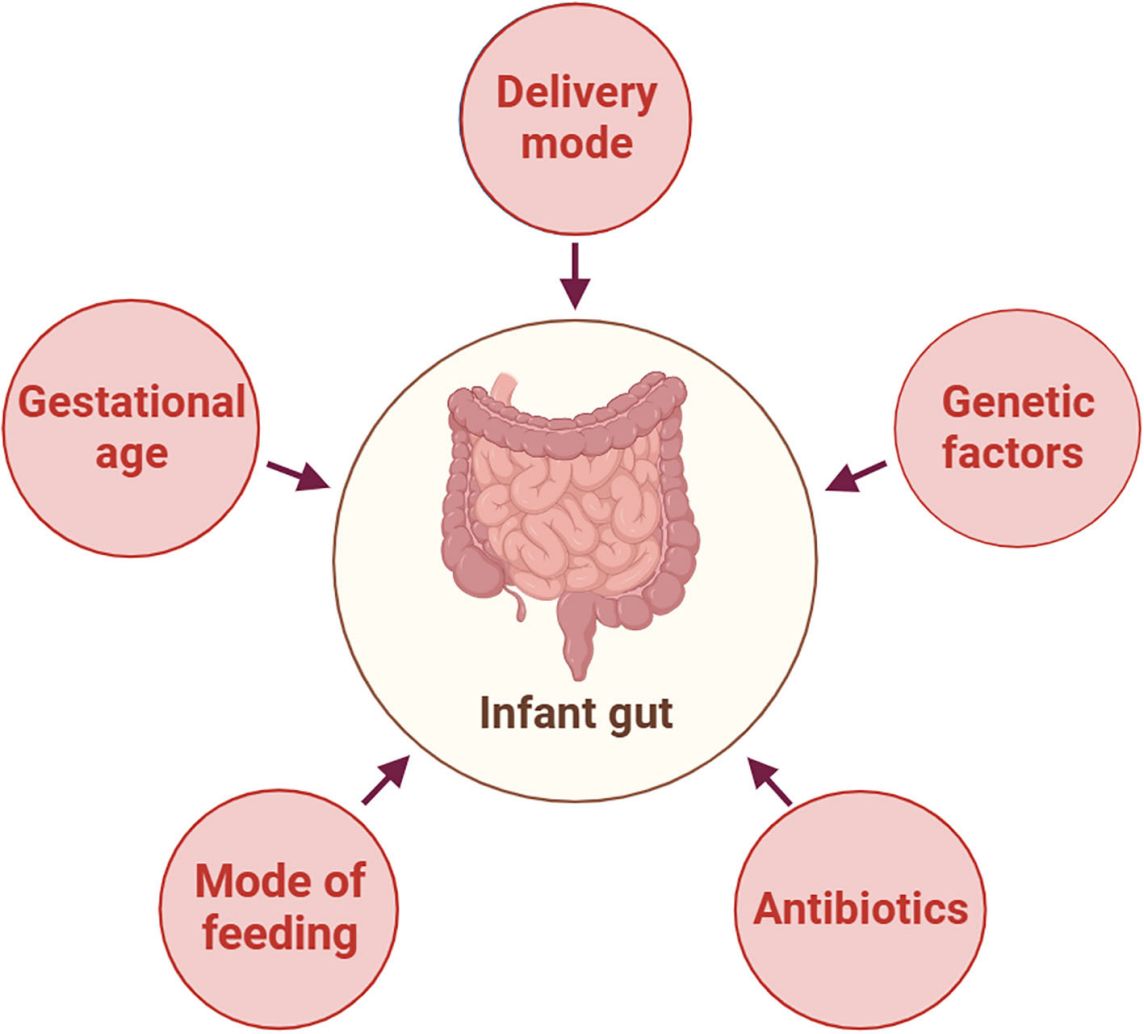
Factors Affecting the Composition of Neonatal Gut Microbiota. This figure was created with BioRender.com.

**Figure 3:**
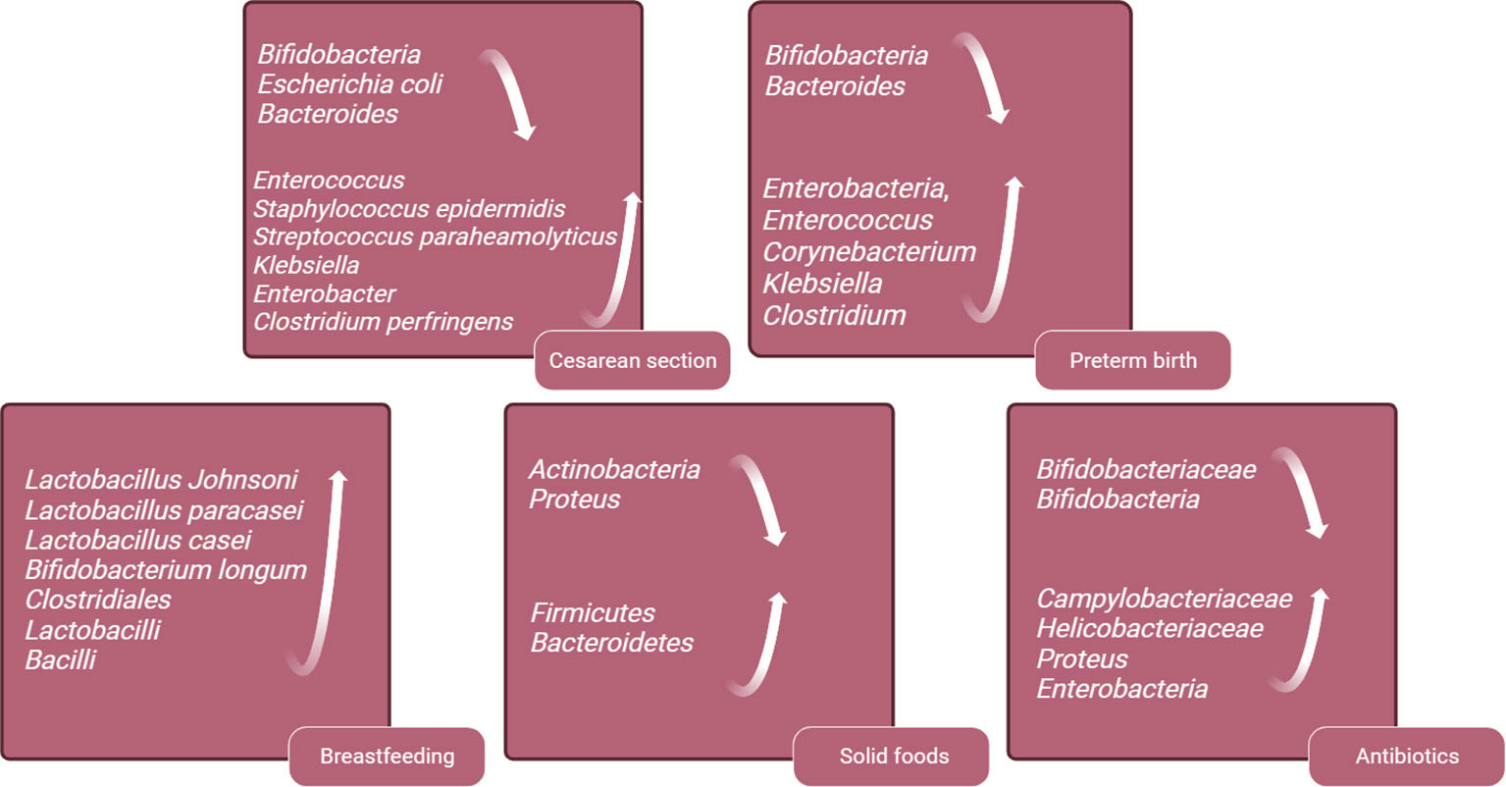
Schematic Diagram of Changes in Infant Gut Microbiota Bacteria Under Various Influencing Factors. This figure was created with BioRender.com.

The early neonatal intestinal flora is influenced by various factors, including maternal and infant-related factors and external environment (O’Neill et al., 2020). This article delineates the influence of factors such as delivery mode and gestational age on the establishment of the infant’s intestinal microbiota (**Figure 2**). A schematic diagram illustrates how bacterial colonization in the infant gut microbiota changes under various influencing factors (**Figure 3**).

### 3.1 Mode of delivery

Intestinal flora plays a pivotal role in the early development of the neonatal immune system and metabolism. The initial colonization of the gut microbiota during the perinatal period represents a critical juncture in the human gut microbiome’s ecological transformation. Fetuses born via cesarean section may experience colonization defects in their intestinal flora, which can have enduring impacts on host’s metabolism and immune development (Olszak et al., 2012). A comprehensive meta-analysis of 23 studies conducted in Northern Ireland has revealed that infants delivered via cesarean section face a 20% higher risk of developing asthma in both childhood and adulthood, in compared to those born through vaginal delivery (Thavagnanam et al., 2008). These findings have been meticulously adjusted to account for potential confounding factors such as maternal smoking, low birth weight, and the duration of breastfeeding. Furthermore, additional studies have noted a heightened susceptibility to allergic rhinitis and atopic dermatitis in children born through cesarean section (Pistiner et al., 2008; Thavagnanam et al., 2008).

In addition, there is a growing focus on exploring alterations in the colonization patterns of gut microbiota and their potential links to long-term risks of obesity and metabolic syndrome. A prospective cohort study found that, even after accounting for influencing factors such as maternal weight and birth weight, children born via cesarean section exhibit a significantly higher incidence of obesity by the age of 3, compared to those delivered vaginally (Huh et al., 2012). In Brazil, another cohort study has reported an increased occurrence of central and peripheral obesity in adults associated with cesarean section (Mesquita et al., 2013). Moreover, infants born via cesarean section have a 23% higher risk of developing type 1 diabetes during childhood (Cho and Norman, 2013). Fetuses born via cesarean section are not exposed to the mother’s vaginal flora but are initially exposed to flora from the mother’s skin or the birthing environment. Early fecal samples from vaginally delivered infants are dominated by *Bifidobacteria*, *Escherichia coli* and *Bacteroides*, accounting for approximately 68.3% of the neonatal gut microbiome. In contrast, infants delivered by cesarean section exhibit depleted populations of these gut microbiome genera, which are replaced by *Enterococcus*, *Staphylococcus epidermidis*, *Streptococcus paraheamolyticus*, *Klebsiella*, *Enterobacter* and *Clostridium perfringens*. These microbiotas are typically found on the skin and in the hospital environment, with 83.7% of infants born via cesarean section carrying opportunistic pathogens during the neonatal period, compared to 49.4% of vaginally born infants. This difference may increase the risk of neonatal infection (Lax et al., 2017; Wampach et al., 2018; Shao et al., 2019).

Cesarean section interrupts the normal transmission route of maternal symbiotic bacteria to infants, potentially increasing the chance of opportunistic pathogen colonization in the intestines. The increasing prevalence of cesarean section in recent years not only increases the risk of pregnancy in the scar area of cesarean section in the second pregnancy, but may also lead to defects in the colonization of fetal intestinal flora. Chu et al. (Chu et al., 2017a) demonstrated that the effect of delivery mode on fetal intestinal flora only lasts for the first 6 months, with no significant difference in flora after this period. Shao et al.(Shao et al., 2019) suggest that although the impact of cesarean section on infants’ intestinal flora diminishes over time, microbiome differences can still be detected up to the age of 1 year. Some studies have even found that differences in gut microbiota between cesarean section and vaginal delivery persist until the age of 7 (Penders et al., 2006; Neu and Rushing, 2011). The short-term or permanent effects of delivery mode on fetal microbiota still require long-term follow-up. Additionally, the routine use of antibiotics during cesarean section is a standard diagnosis and treatment process, so it remains uncertain whether antibiotics, as an interfering factor in comparing intestinal flora between vaginal and cesarean-born infants, impact the results.

The study conducted by Santos et al. presents a noteworthy finding that challenges the conventional understanding of the relationship between the maternal vaginal microbiome and the early development of the infant gut microbiome(Dos Santos et al., 2023). Traditionally, it has been believed that infants acquire their initial microbial communities during birth, particularly through exposure to maternal vaginal flora. However, the study’s results suggest otherwise, indicating that the composition of the maternal vaginal microbiome may not have a significant impact on the early years of the infant gut microbiome. This finding sparks further inquiry into the multifaceted factors that shape early gut microbiota and their implications for child health and development.

### 3.2 Gestational age

Gestational age at delivery is another crucial factor that impacts the establishment of intestinal flora in infants. Gestational age at birth significantly influences the formation and development of the intestinal microbiota, particularly in preterm infants. Research indicates that gestational age at birth plays a crucial role in shaping the structure and diversity of the intestinal microbiota. Gregory et al. (Gregory et al., 2016) proposed that gestational age at birth determines the trajectory of microbiota development within the first three weeks after birth. Chernikova et al.(Chernikova et al., 2018) demonstrated that gestational age at birth has a notable impact on the composition of the intestinal microbiota, with extremely preterm infants having a Simpson index of 0.35, compared to 0.65 in preterm infants. Furthermore, Drell et al. (Drell et al., 2014) emphasized a positive correlation between corrected gestational age and microbiota diversity, highlighting the role of gestational age in the evolution of the microbiota during early childhood.

Many diseases in premature infants are closely associated with their intestinal flora, such as necrotizing enterocolitis (NEC) (Aguilar-Lopez et al., 2021). Impaired composition and functionality of the intestinal flora have a direct impact on the health and potential complications of preterm infants. A better understanding of the differences in intestinal flora between preterm and full-term infants can help address the intestinal flora deficiencies in preterm infants and reduce related diseases.

In comparison with full-term infants, preterm infants have lower intestinal flora diversity, delayed colonization of *Bifidobacteria* and *Bacteroides*, and an increase in opportunistic pathogens like *Enterobacteria*, *Enterococcus*, *Corynebacterium*, *Klebsiella* and *Clostridium* (Mai et al., 2013; Arboleya et al., 2015). In addition, studies have found that the levels of short-chain fatty acids, which are metabolites products of the flora, are lower in the gut of preterm infants compared to full-term infants (Bergström et al., 2014). Premature infants exhibit significant differences in their intestinal microbiota compared to full-term infants. These distinctions may render premature infants more susceptible to microbial imbalances, leading to alterations in metabolic byproducts and potential ramifications for immune system development, as well as the risk of related diseases. These disparities are often attributed to the influence of various medical factors that premature infants are frequently exposed to, including cesarean section delivery, lack of breastfeeding, mechanical ventilation, and the frequent use of antibiotics. These factors can contribute to variations in the composition of the intestinal microbiota(Cuna et al., 2021). To improve the deficiency of intestinal flora in premature infants, this review will provide the following information.

### 3.3 Feeding mode

In recent years, the research and findings have primarily focused on the influence of maternal symbiotic flora on neonatal intestinal flora colonization but have often overlooked the impact of acquired breastfeeding. Breast milk is the best source of nutrition for infants, and the World Health Organization advocates exclusive breastfeeding until six months of age, with complementary foods gradually added, and breastfeeding continuing for at least two years (Binns et al., 2016).

When compared to formula-fed infants delivered vaginally, exclusively breastfed infants have a higher presence of probiotics in their gut, including *Lactobacillus Johnsoni*, *Lactobacillus paracasei*, *Lactobacillus casei* and *Bifidobacterium longum* (Bäckhed et al., 2015). The imbalance of intestinal flora in infants resulting from cesarean delivery can be corrected through subsequent breastfeeding. At six months postpartum, the composition and structure of the intestinal microbiome in infants delivered via cesarean section differ from those delivered vaginally, with fewer *Bacteroides* and *Shigella genera*, and more *Klebsiella*, *Veillonella* and *Enterococcus faecalis.* The differences in intestinal microbiome caused by delivery the mode of delivery can be mitigated by acquired exclusive breastfeeding (Liu et al., 2023). Breastfeeding can reduce the incidence of NEC and sepsis in preterm infants (Quigley et al., 2018). Breastfeeding is vital for the establishment of intestinal flora in preterm infants. Studies have shown that preterm infants who are breastfed have increased gut microbiome alpha diversity compared to formula feeding (Cong et al., 2016). There are more *Clostridiales*, *Lactobacilli* and *Bacilli* in the intestines of premature infants who are breastfed compared to formula feeding and donor milk feeding (Cong et al., 2017). Breast milk is beneficial to the intestinal microbiome development of premature infants and increases the microbial diversity in early life, and it is recommended to support breast-feeding for premature infants after birth.

The weaning process represents the final trajectory of infant gut microbiota formation. In this process, diet plays a crucial role in regulating the microbial community (Kumbhare et al., 2019). Before weaning, the gut microbiota’s functional repertoire primarily involves gene expression related to the utilization of lactose (Tanaka and Nakayama, 2017). However, with the introduction of solid foods, the gene expression of the gut microbiota begins to increase in functions such as carbohydrate utilization, vitamin synthesis, and xenobiotic degradation. This transitional process is consistent with the rapid development of the immune system and changes in dietary intake.

The introduction of solid foods is another key factor in regulating the composition of the gut microbiota and marks the beginning of its maturation phase. This maturation of the microbial community is a gradually evolving process that ultimately leads to a more diverse and stable microbial profile (Davis et al., 2020). The introduction of solid foods triggers changes in the infant gut microbiota, and the supplementation of new nutrients increases the alpha -diversity of the gut microbiota, with *Firmicutes* and *Bacteroidetes* replacing *Actinobacteria* and *Proteus* as dominant phyla (Fallani et al., 2011; Koenig et al., 2011; Martin et al., 2016). Additionally, the total amount of short-chain fatty acids increases, with a significant rise in butyrate levels. The introduction of solid foods also increases the intake of plant-derived polysaccharides. During this stage, the composition of *Bifidobacterium* gradually shifts towards strains capable of metabolizing plant-derived polysaccharides, such as *Bifidobacterium longum* and *Bifidobacterium adolescentis* (O’Callaghan and van Sinderen, 2016). Simultaneously, *Bifidobacterium bifidum* redirects its metabolic capacity from human milk oligosaccharides (HMOs) towards mucin degradation.

Increased protein intake leads to an increase in the abundance of *Lachnospira* and a decrease in *Bifidobacterium* (Laursen et al., 2016). On the other hand, high fiber intake is associated with changes in the genus *Prevotella*. The addition of solid foods also marks the beginning of the transition of the infant gut microbiota structure towards an adult-like structure, with a microbial community becoming more complex to adapt to the plant-derived polysaccharides present in the adult diet, promoting mutualistic symbiosis between the host and microbiota (Koenig et al., 2011). This process continues until the age of two when the gut microbiota structure of infants reaches a stable state similar to that of adults.

### 3.4 Antibiotics

#### 3.4.1 Effects of antibiotics on infant intestinal flora

Perinatal exposure to antibiotics has a significant impact on the early establishment of gut microbiota in offspring and increases the risk of childhood asthma, allergies, and obesity (Cox et al., 2014; Leclercq et al., 2017). Antibiotics affect an infant's gut microbiota in two ways: (1) Antibiotics can reach the fetal bloodstream through the umbilical cord. (2) Antibiotics alter the maternal vaginal and intestinal microbiome, leading to vertical transmission of infant intestinal flora abnormalities. A study on the effects of intrauterine antibiotic use on neonatal intestinal microbiota found that infants exposed to intrauterine antibiotic had decreased levels of Bifidobacteriaceae and an increased the proportion of potentially pathogenic microorganisms, including Campylobacteriaceae or Helicobacteriaceae (Nogacka et al., 2017). Another similar study found that infants treated with antibiotics during delivery had lower gut bacterial diversity, a decreased relative abundance of actinomycetes (especially Bifidobacteriaceae), and a higher relative abundance of Proteus in the gut microbiota (Zimmermann and Curtis, 2020). In full-term infants, the administration of antibiotics within a few hours after birth can reduce the level of Bifidobacteria in the gut and increase the level of Enterobacteria (Arboleya et al., 2015). The use of antibiotics during and after delivery can significantly affect the intestinal flora of infants, and it is recommended to avoid antibiotics unless medically necessary to reduce infants' exposure to antibiotics.

Given that premature infants tend to have relatively fragile immune systems and are more susceptible to infections, antibiotics are commonly administered for therapeutic purposes (Groer et al., 2014; Yasmin et al., 2017). In fact, antibiotics rank among the most frequently prescribed medications in the Neonatal Intensive Care Unit (NICU). However, the use of antibiotics presents a double-edged sword. While antibiotics can reduce the mortality rate associated with infectious diseases in premature infants, they often disrupts and disturbs the composition of the intestinal microbiota, consequently increasing the risk of various diseases (Morowitz et al., 2022). Additionally, research indicates that premature infants, particularly those with extremely low birth weights, face an increased risk of developing conditions like sepsis and NEC as a result of prolonged exposure to antibiotics (Cantey et al., 2018; Vatne et al., 2023).

#### 3.4.2 Influence of timing of antibiotic application in cesarean section

As the prophylactic application of antibiotics to pregnant women during cesarean section is a routine obstetrical diagnosis and treatment process, antibiotics that can cross the placenta may reach the fetus through the placental barrier. In order to prevent intrauterine exposure of newborns to antibiotics, antibiotics are administered after umbilical cord cutting in some countries to minimize the exposure of newborns to antibiotics (Stinson et al., 2018). It is worth to study whether the timing of antibiotics for cesarean section prophylaxis can affect the gut microbes of infants or not, thus revealing the influence of antibiotic exposure before umbilical rupture to fetal gut flora. Relevant studies have compared the use of antibiotics before skin incision and after the umbilical cord is clamped in the gut microbiome composition of full-term infants born via elective cesarean section. Surprisingly, there were no significant difference in the gut microbiome composition observed at 9 months of age, and the potential long-term effects of antibiotics cannot be ruled out based on this study (Kamal et al., 2019). Similar results were found in another study, which reported no difference in intestinal microbiota composition between 10 and 9 months after birth (Dierikx et al., 2022). Prenatal antibiotic exposure in infants born via cesarean section does not appear to affect early-life microbiome development, but long-term follow-up is needed.

### 3.5 Genetic factors and gender

There is growing evidence that genetic factors influence the gut microbiota of infants. The similarity of intestinal flora between identical twins is higher than that between fraternal twins, indicating that genetic factors have an impact on fecal microbial community (Stewart et al., 2005). A study has proved that there are differences in the gut microbiome in males and females, with male babies having a lower gut microbiome diversity index at birth, while female babies have a higher gut microbiome diversity index, and female babies have a higher abundance of *Clostridium difficile* and lower abundance of *Enterobacterium* than male babies (Cong et al., 2016). Another similar study demonstrated that the relative abundance of *Bacteroides* in the intestinal flora of 3-month-old male infants was lower than that of female infants (Kozyrskyj et al., 2016). Genetic factors may play a potential role in the establishment of infant intestinal microbiota, and the mechanism of sex difference on intestinal microbiota community is still unclear.

## 4 Interventions to compensate for the defects of neonatal intestinal flora colonization

### 4.1 “Vaginal seeding”

In recent years, there has been growing interest in a research topic called ‘vaginal seeding’ as a way to help newborns born by cesarean section establish a healthy gut microbiome. This involves swabbing newborns with gauze containing maternal vaginal fluid, starting from the lips and proceeding to different parts of the body to mimic the natural birth process. The idea is to introduce beneficial vaginal microbes to cesarean-born babies, with the aim of making their gut flora more similar to that of babies born through vaginal delivery. Studies have shown that this ‘vaginal seeding’ can indeed help cesarean-born babies establish a gut microbiome that resembles that of babies born vaginally.

However, it’s important to note that current research has primarily focused on short-term effects, and we lack a clear understanding of the long-term consequences. There is also a concern about the potential risk of neonatal infection due to the introduction of vaginal bacteria during the ‘vaginal seeding’ process. Therefore, caution is needed, and more clinical studies are necessary to ensure the safety of this practice. If this practice is proven to be beneficial and safe, it’s important to assess its acceptance among expectant mothers.

Furthermore, researchers have explored different methods of introducing maternal vaginal microbes to cesarean-born babies. For example, a study by Wilson et al. (Wilson et al., 2021) attempted to give cesarean section newborns an oral solution containing maternal vaginal microbial. Surprisingly, the results showed no significant difference in the composition and development of the babies’ intestinal flora compared to those who received a standard oral saline solution. This suggests that the method of introducing mother’s vaginal microbes may not be as effective in reconstructing the gut flora of cesarean-born babies as previously thought. More experiments are needed to verify the impact of various seeding methods on the reconstruction of the newborns’ gut microbiome.

### 4.2 Maternal intestinal flora transplantation

It has been reported that a newborn’s intestinal flora is primarily derived from the mother’s intestinal flora, with seeding of the maternal vaginal flora playing a secondary role in the colonization of the infant’s fecal flora (Sakwinska et al., 2017; Turunen et al., 2021). Ferretti P et al. (Jiménez et al., 2005) demonstrated that maternal intestinal, vaginal, skin and oral flora all contributed to the establishment of infant’s intestinal flora. Maternal fecal flora makes the most significant contribution to the infant’s intestinal flora, accounting for 22.1% of the infant’s intestinal microflora, followed by vaginal flora (16.3%). However, the contribution of vaginal flora species to infant feces diminishes over time. If vaginal microbiota isn’t the primary source of intestinal microbiota in infants, then the effectiveness of “vaginal seeding” in restoring intestinal microbiota colonization defects in newborns born by cesarean section is questionable. It is suspected that fecal microbiota transplantation (FMT) may correct the imbalance of intestinal microbiota in infants born by cesarean section. In an experimental setting, within 10 hours of birth, the mother’s fecal bacterial solution was mixed with breast milk and fed the cesarean-born infants, who did not experience adverse reactions. FMT treatment not only restored the intestinal microbiome of the cesarean-born infants but also led to the development of their gut microbiota being more similar to that of vaginally delivered infants. The microbiota of infants delivered by cesarean section with FMT in the early postpartum period was different from that of those delivered vaginally. However, from day 7 onwards, the gut microbiota of infants treated with FMT resembled that of infants delivered vaginally. Furthermore, FMT reduced the presence of intestinal opportunistic pathogens such as *Enterococcus*, *Enterobacter*, and *Klebsiella* in cesarean newborns (Korpela et al., 2020). Maternal intestinal flora transplantation has shown significant advantages in rectifying intestinal flora deficiencies in neonates born by cesarean section. Therefore, it may be feasible to consider the transplantation of maternal intestinal flora. However, the feasibility, safety, and clinical applicability of methods like ‘vaginal seeding’ and fecal bacteria transplantation still require further evaluation.

Further evaluation is needed to determine the feasibility and clinical applicability of ‘vaginal seeding’ and fecal bacteria transplantation as therapeutic methods to compensate for congenital defects in intestinal flora colonization.

### 4.3 Probiotics and prebiotics

Probiotics are live microorganisms that provide benefit to the host by colonizing the human body and changing the composition of the flora in a certain part of the host. The deficiency of newborn intestinal flora can be restored by probiotics. For premature infants, probiotics can enhance intestinal barrier function by regulating flora structure, reduce the colonization and migration of pathogenic bacteria, and promote the development and functional maturation of intestinal immune cells in newborns, thus reducing the incidence and mortality of NEC (Dermyshi et al., 2017; Gopalakrishna et al., 2019; Morgan et al., 2020). After oral administration of probiotics, preterm infants have a higher relative abundance of *Bifidobacterium* and *Enterobacterium*, and a lower relative abundance of *Escherichia coli*, *Enterococcus* and *Klebsiella* in the gut, and a lower incidence of NEC (Patole et al., 2014; van Best et al., 2020). Some studies have shown that early probiotic supplementation with *Lacticaseibacillus rhamnosus GG* (LGG) and *Bifidobacterium animalis* can affect the colonization of potential pathogens in infant intestines (Hui et al., 2021). Probiotics showed great benefit in restoring early intestinal flora colonization defects in preterm infants. It is recommended to apply probiotics in the early life of premature infants to prevent and treat intestinal flora defects and related diseases in premature infants.

Antibiotics can cause defects in infants’ gut microbiota and are associated with health problems in later life (Hviid et al., 2011; Metsälä et al., 2013; Azad et al., 2014; Chu et al., 2015). Probiotic supplementation can reshape antibiotic-treated intestinal microbiota disturbances in mice (Grazul et al., 2016). In the study (Zhong et al., 2021), it was found that exposure to piperacillin-tazobactam reduced the richness of the gut microbiota in full-term infants and may interfere with *Bifidobacterium* and *Lactobacillus* reproduction. It was also found that simultaneous treatment with probiotics and antibiotics was beneficial to the gut microbiota and could lead to an increase in *Bifidobacterium*. When supplementation with probiotics was delayed, there appeared to be no effect and no benefit from intervening in the gut microbiota disrupted by antibiotics. This study emphasizes that probiotics should be taken at the same time as antibiotics in the treatment of infants with intestinal flora disorders caused by antibiotics, and its therapeutic effect will be greater.

The alpha diversity of intestinal flora was significantly lower in newborns delivered by cesarean section compared to those born vaginally. However, after probiotic supplementation, the alpha diversity and beta diversity of intestinal flora in infants delivered by cesarean section become similar to those delivered vaginally. Additionally, there was a significant increase in the abundance of *Lactobacillus* and *Bifidobacterium* in the gut (Yang et al., 2021). The effect of probiotics on intestinal microbiota of newborns delivered by cesarean section also depends on the feeding mode of infants. In comparison to formula milk powder, breastfeeding led to an increase in intestinal *Bifidobacterium* and a decrease in *Proteus* and *clostridium* in cesarean-born infants (Korpela et al., 2018). Therefore, it is recommended to supplement probiotics to newborns delivered by cesarean section, and the promotion of infant breastfeeding is encouraged.

Overall, when compared to non-supplemented formulas, these prebiotic-supplemented ones increase the softness of stools (Skórka et al., 2018). They might also be able to decrease the incidence of enteric infections and diarrhea, reduce eczema, and increase *Bifidobacteria* counts. In premature infants, researchers have extensively studied various combinations of non-human milk galacto-, fructo-, and acidic oligosaccharides. These prebiotic mixtures have been found to modify the fecal microbiome, lower fecal pH, enhance gastric motility, reduce feeding intolerance, and increase fecal sIgA (Westerbeek et al., 2013). However, despite these positive effects, there is currently no related research on whether prebiotics can restore the colonization defects in the gut microbiota of newborns delivered by cesarean section, and this requires further investigation.

## 5 Conclusions and perspectives

The question of whether fetal intestinal microbiota colonization occurs *in utero* or postnatally remains a subject of ongoing research with no definitive consensus. Traditional beliefs held that the fetal environment was sterile, with the infant’s gut primarily being colonized following birth through exposure to maternal and environmental microorganisms. However, recent investigations have introduced the possibility of some degree of microbial exposure or colonization *in utero*. Despite this, the extent and clinical significance of such prenatal microbial colonization remain subjects of investigation. Several studies have reported the presence of microbial DNA in amniotic fluid, placenta, and meconium (the initial fecal material passed by a newborn), sparking inquiries into the potential for prenatal microbial colonization. Yet, questions persist about the origins and roles of these microorganisms in the developing fetus. To gain a more comprehensive understanding of the timing and mechanisms underlying the establishment of the infant gut microbiota and the potential occurrence of prenatal colonization, further clinical studies are essential.

In shaping the infant’s gut microbiota, the review underscores the crucial role of the maternal-infant symbiotic relationship. Various microorganisms from different maternal body sites contribute to the development of the infant’s intestinal microflora, with the maternal gut microbiota making the most significant contribution. The infant’s gut microbiota undergoes multiple stages of change during the early period. It initially consists of aerobic or facultative anaerobic bacteria, gradually transitioning to anaerobic bacteria, such as *Bifidobacterium*, *Clostridium*, and *Bacteroides*. Understanding these transitions provides valuable insights into the formation of the infant’s gut microbiota.

While the colonization of the infant’s gut by maternal vaginal, oral, and skin flora is relatively short-lived, it has prompted discussions about the feasibility and potential benefits of the ‘vaginal seeding’ strategy. To determine when the infant’s gut microbiota tends to stabilize, resembling that of adults, further in-depth research is needed. This understanding contributes to a better comprehension of the evolution of the intestinal microbiota.

Future studies should delve deeper into the origins and dynamic changes of the infant’s gut microbiota to elucidate the potential impact of early microbial colonization on the health of children and adults. Additionally, there is a need for further research to explore the mechanisms and potential significance of prenatal microbial colonization.

Intestinal flora colonization is a complex process influenced by various factors, including delivery mode, gestational age, feeding mode, and antibiotic usage. The impact of delivery mode on newborn intestinal flora colonization is significant. Non-cesarean section indications should be advocated, and vaginal delivery should be selected as far as possible to avoid congenital colonization defects of infant intestinal flora. Understanding the impact of cesarean section on the neonatal gut microbiome and subsequent health outcomes is crucial. These findings shed light on the potential risks associated with cesarean section. Moreover, the need for long-term follow-up studies cannot be overstated. Such studies would allow us to gain a more comprehensive understanding of whether the effects observed persist over time or gradually diminish. This knowledge is crucial for healthcare professionals and parents alike. Exploring potential intervention measures to bridge the gap in gut microbiota between cesarean-born and vaginally born infants is another avenue for future research. This could include methods to promote the colonization of cesarean-born infants’ gut with flora closer to that of vaginally born infants or strategies to maintain a healthy balance of gut microbiota after cesarean section.

Preterm infants are typically delivered by cesarean section, and most receive antibiotic treatment while lacking the breastfeeding. Prolonged exposure to the hospital environment can exacerbate colonization defects in the intestinal flora of preterm infants. Future research needs to delve deeper into the diversity of the gut microbiota in preterm infants, including differences compared to full-term infants, in order to comprehensively understand its formation and evolution. Additionally, there is a need to enhance investigations into the connections between gut microbiota and diseases relevant to preterm infants, such as necrotizing enterocolitis, to determine the specific impact of the microbiota on these conditions, thus providing a basis for future prevention and treatment. It’s crucial not only to comprehend the composition of the gut microbiota but also to conduct in-depth research into their functions in metabolism, the immune system, and other physiological aspects to reveal more profound health associations. Long-term follow-up studies are indispensable to understand the extended development of the gut microbiota in preterm infants and their potential long-term health consequences, especially in adulthood, regarding potential risks related to the gut microbiota. Future research should focus on gaining a more comprehensive understanding of the formation and influence of the gut microbiota in preterm infants, with the goal of improving their health, reducing disease risks, offering more effective intervention measures, and enhancing their quality of life.

The use of antibiotics during the perinatal and postnatal periods can impact a baby’s gut microbiota. It is standard practice to administer prophylactic antibiotics to pregnant women during cesarean section. In order to minimize early antibiotic exposure for newborns, some guidelines recommend using antibiotics to prevent infection after umbilical cord cutting. Current studies suggest that the timing of antibiotic administration does not appear to affect the establishment of the baby’s gut microbiota. Breastfeeding is highly beneficial for establishing the intestinal flora in infants. It is recommended for newborns, and even in situations where breastfeeding is not feasible, it remains valuable. Breastfeeding contributes to the development of the intestinal microbiome of the intestinal microbiome in premature infants and enhances the microbial diversity in early life.

There are various perspectives on the source of neonatal intestinal flora colonization, including discussions about which components of the mother’s flora play a more beneficial role in compensating for congenital neonatal intestinal flora colonization defects. Debates persist regarding the feasibility of practices such as ‘vaginal seeding’, maternal fecal flora transplantation, and the choice between single-flora or multi-flora transplantation methods. More clinical studies are needed to validate these approaches. Breastfeeding, along with the use of probiotics and prebiotics, may have a beneficial effect in addressing colonization defects in the gut microbiota of preterm infants, newborns delivered by cesarean section, or infants affected by disorders related to gut microbiota establishment. Future research could consider gut microbes as a potential intervention to promote infant health.

## Author contributions

GM: Writing – original draft. YS: Writing – original draft. LM: Writing – original draft. HF: Writing – original draft. XT: Writing – original draft. HL: Writing – original draft. DW: Writing – original draft. JZ: Writing – original draft. XX: Writing – review & editing.
